# Neural Networks Predicting Microbial Fuel Cells Output for Soft Robotics Applications

**DOI:** 10.3389/frobt.2021.633414

**Published:** 2021-03-04

**Authors:** Michail-Antisthenis Tsompanas, Jiseon You, Hemma Philamore, Jonathan Rossiter, Ioannis Ieropoulos

**Affiliations:** ^1^ Bristol BioEnergy Centre, Bristol Robotics Laboratory, Frenchay Campus, University of the West of England, Bristol, United Kingdom; ^2^ SoftLab, Department of Engineering Mathematics, University of Bristol, Bristol, United Kingdom

**Keywords:** microbial fuel cells, soft robotics, neural network, nonlinear autoregressive network, robotic control

## Abstract

The development of biodegradable soft robotics requires an appropriate eco-friendly source of energy. The use of Microbial Fuel Cells (MFCs) is suggested as they can be designed completely from soft materials with little or no negative effects to the environment. Nonetheless, their responsiveness and functionality is not strictly defined as in other conventional technologies, i.e. lithium batteries. Consequently, the use of artificial intelligence methods in their control techniques is highly recommended. The use of neural networks, namely a nonlinear autoregressive network with exogenous inputs was employed to predict the electrical output of an MFC, given its previous outputs and feeding volumes. Thus, predicting MFC outputs as a time series, enables accurate determination of feeding intervals and quantities required for sustenance that can be incorporated in the behavioural repertoire of a soft robot.

## 1 Introduction

Modern society is both driven by and highly dependent on technology. One significant technological field that has entered many aspects of our everyday lives is robotics. Robots are often required to operate autonomously in environments that are too dangerous or distant for humans to occupy, such as in the open ocean or on other planets. This can lead to the robot becoming a pollutant in the event of a breakdown in the field, if it cannot be recovered. The continuously faster incorporation of innovations to the market and the heavy duty use of robotics, render a great amount of devices outdated, unusable or problematic. As a result, there is a vast amount of non-recyclable and sometimes toxic parts and materials that need to be disposed. This leads to significant environmental issues that further complicate climate change.

Robots that are no longer functional, leave a variety of components useless, like rigid parts (metal and plastic) for the body, electronics for controlling, motors for movement and batteries for energy storage. Consequently, an innovative, possible countermeasure for this is the design of biodegradable and bio-compatible soft robotics (Rossiter et al., 2016). Soft bodied robots are additionally useful for interacting benignly with natural environments and organisms due to their structural compliance. Tough and elastic soft materials have the potential to reduce damage incurred by the robot when interacting with unpredictable, open environments. The development of smart materials can enable the fabrication of robots with biodegradable and bio-compatible components for all different parts (Philamore et al., 2016; Rossiter et al., 2017), like body parts, movement and control. So, when the robot is released in the environment it will degrade and not burden the climate balance, or have negative toxic effects to animals or plants.

The part of the robot that will be considered here is the one responsible for providing the energy (for movement, sensing, control etc). In specific, Microbial Fuel Cells (MFCs) (Santoro et al., 2017) are proposed as their long history of research permitted the transit of construction materials from toxic and expensive to biodegradable and cheap. Namely, they have been previously build by all kinds of bio-combatible materials (Winfield et al., 2013a; Winfield et al., 2013b; Winfield et al., 2015a; Winfield et al., 2015b), i.e. lanolin, gelatine, egg and paper. Moreover, MFCs are not toxic, as they accommodate anaerobic bacteria that release electrons as a byproduct of their metabolism on organic matter. These electrons are collected to form an electrical current that can be stored or used on-the-fly to move a soft robot (Ieropoulos et al., 2003; Ieropoulos et al., 2005; Ieropoulos et al., 2010). Numerous examples of MFCs made from soft materials exist (Winfield et al., 2014; Slate et al., 2019), making them highly suitable for use in soft robots. These soft and bio-compatible sources of power therefore show great potential for use in robots deployed safely and benignly in natural environments, for purposes such as environmental monitoring, when compared to conventional batteries comprised of rigid and toxic components.

MFCs and soft robots pose a common challenge in that it is difficult to predict the output behaviour for a given input using conventional model based approaches. In the case of soft robot control, model-based control theory developed for rigid body robots is often poorly suited due to the difficultly in defining exact kinematic and dynamic models of highly non-linear and under-actuated systems. Complex shapes and smart materials widely used in soft robotic sensors and actuators make it difficult and to define constitutive equations of the materials. Materials that exhibit other mechanical non-linearities such as creep, hysteresis and non-stationarity, further increasing the difficulty in modelling soft robots and sensors for control purposes. A widely used approach is therefore to use bio-inspired learning algorithms for control of soft robots (Wilson et al., 2016; Thuruthel et al., 2018). Strategies include learning the inverse kinematics of soft actuators (Thuruthel et al., 2016), predictive control (Thuruthel et al., 2019), and mapping sensor outputs to real world values (Pastor et al., 2019).

Moreover, despite the plethora of advantages that MFCs have, one major limitation is the low predictability of their performance, the differentiation of their outputs and the significance of some environmental conditions to their efficient performance (Picioreanu et al., 2010). The MFC electrical output is determined by a large number of constant and time dependent parameters, many of which are difficult to control due to their biological (e.g. bio-film growth) or environmental nature (e.g. temperature). While there are several works that simulate the behaviour of MFCs (Picioreanu et al., 2008; Pinto et al., 2010; Tsompanas et al., 2017a; Tsompanas et al., 2018), a more dynamic modelling tool is required to tackle the non linear behaviour observed in MFCs. Inspired by this, we propose the training and utilization of Nonlinear Autoregressive Networks with exogenous inputs (NARX) (Lin et al., 1996; Menezes and Barreto, 2008) to predict a time series of the outputs of MFCs that can be used on biodegradable soft robotics. Thus, we can anticipate the intervals that refueling is needed and maximize the capabilities of these soft robots.

The predictive modelling proposed will enable scheduling of feed-times with reduced energy spent on sampling the MFC voltage output. For example, a robot may be able to estimate at what point within the next week it will need to refuel. In soft robots this may be coupled with ANN control systems making the realisation of soft, biomimetic and environmentally-friendly robots more viable.

## 2 Previous Work

MFCs have been used as a bio-inspired source of electrical power in the pioneering EcoBot robot series; autonomous mobile robots powered by an on-board bank of MFCs (Ieropoulos et al., 2010). More recently, MFC power sources have been used in biomimetic robots such as the Row-bot, an insect-inspired swimming robot with a single MFC ‘artificial stomach’ as its sole source of power (Philamore et al., 2015b). The powered actuation of the Row-bot includes the operation of a soft-robotic mouth which is used to energy-autonomously ‘feed’ the artificial stomach with fresh-fluid from its surroundings. The simple control system of the Row-bot uses the threshold voltage of a storage capacitor to determine the timing of the Row-bot’s behavioral cycle to swim and feed. This behavioural control could be greatly improved by using machine learning (ML) tools to predict the temporal voltage profile for a given input volume and thereby determine the optimum feeding interval for maximising the energy stored per batch of food and the robot’s powered activity. This is of particular importance due to the extreme low energy budget of a robot powered by a single MFC and the resulting critical need to run as efficiently as possible to remain operational. The viability of miniature MFC-powered robots may be greatly improved by employing intelligent feeding control algorithms such as Artificial Neural Networks (ANNs). The use of ANNs and other ML algorithms are a promising technology to enable mobile MFC-powered, soft swimming robots, deployed in the field in unknown aquatic environments to learn and adapt to their surrounding environments.

Other examples of MFCs as power sources for soft robots include the use of ionic polymer metal composites (IPMCs) as both low-voltage soft robotic actuators and, in a novel application, as the ion exchange membrane of an MFC in an MFC-powered tadpole-inspired soft robot (Philamore et al., 2015a). This work demonstrated the potential to build miniature bio-powered soft robots by using multi-functional smart materials for power and actuation. However, a bottle-neck with this technology is the extremely low power generated by such miniaturized MFC systems, which may be more efficiently managed using predictive ML. The combination of bio-inspired ANNs for power management of a bio-hybrid source of power to drive bio-mimetic soft mechanisms therefore holds great potential for the development of robots with robust morphological and behavioural adaptation to the environment, analogous to a natural organism.

The behaviour of MFCs in general have been previously predicted by ML methods. Specifically, voltage outputs, Chemical Oxygen Demand removal rates, Coulombic efficiency and other characteristics of MFCs have been approximated by multilayer perceptron ANNs (Tardast et al., 2012; Tardast et al., 2014; Jaeel et al., 2016; Ismail et al., 2017; Lesnik and Liu, 2017; Tsompanas et al., 2019; de Ramón-Fernández et al., 2020), multi-gene genetic programming (Garg et al., 2014), adaptive neuro-fuzzy inference systems (Esfandyari et al., 2016), nonparametric Gaussian process regression models (He and Ma, 2016) and support vector regression forward and inverse model (Wang et al., 2018). Despite the increasing popularity of modelling and optimizing MFC outputs with ML (Ghasemi et al., 2020; Jadhav et al., 2020), implementing time series analysis is not that frequent. For instance, time parameters were used as inputs for neural networks in the study of MFCs (Garg et al., 2014; Jaeel et al., 2016; Ismail et al., 2017); however, this methodology has some limitations. In specific, in time series analysis, a few past states of the system are more informative than the time past from the moment *t* = 0. Thus, several modifications of neural networks, like convolutional and recurrent neural networks and NARX, have proved to be more efficient in time series prediction. The use of NARX networks is ideal for time series analysis, and have been used for smart biosensing with MFCs (Feng et al., 2013).

One drawback in all current systems is their reliance on silicon computation. The resilience and questionable biocompatibility of conventional computational systems limits the deployment of MFC-based soft robots in real-world open environments. Unconventional or non-silicon computation has been previously reported (Teuscher, 2014) and recently, significant advances in soft materials computation systems and organic electronics, have opened the way for truly embedded computation and learning within the body of the robot. For example, Rothemund et al. (2018) developed a soft valve capable of controlling worm-like robot. These valves were subsequently composed to form elementary electronic components, including two-bit adders and shift registers (Preston et al., 2019). Fluidic controls have also been integrated into origami structures (Li and Wang, 2015), and mechanical logic gates (Song et al., 2019). Complex digital and analog computing and control have been demonstrated in the soft matter computer (SMC) (Garrad et al., 2019) which can be integrated directly into the body of a soft (or MFC-based) robot with only minimal modification. The SMC is driven by fluidic energy (available as a by-product in many MFC systems) and couples electrofluidic ‘transistors’, a range of ‘receptor’ sensors, and soft actuators. Turing completeness with cellular automata has been reported using MFCs that make reference to additional pins - akin to transistors - which has been shown to solve the Game of Life algorithm, as an example where MFCs can be used as information processing units (Tsompanas et al., 2017b). Finally, advancements in materials science have made bacterial communities, such as Shewanella oneidensis, integration into organic electronics (PEDOT-PSS) possible, resulting in organic microbial electrochemical transistors (Méhes et al., 2020), i.e. the building blocks of computation. This provides the complete toolkit of components needed to implement simple processes within soft-bodied MFC-powered robots.

## 3 Nonlinear Autoregressive Network With Exogenous Inputs

Some applications of NARX networks are predictors, nonlinear filters or models of nonlinear dynamic systems. NARX network is a recurrent dynamic network, equipped with feedback loops that can include several layers. The NARX model is frequently utilized for modeling time series (Lin et al., 1996; Menezes and Barreto, 2008). It can be mathematically represented by the following equation:

$$y\left(n+1\right)=f\left[y\left(n\right),y\left(n-1\right),...,y(n-d_{y}\mathrm{+1),}u\left(n\right),u\left(n-1\right),...,u(n-d_{u}\mathrm{+1)}\right]$$ (1)

where *y*(*n*) and *u*(*n*) are the output and the inputs, respectively, of the network at the discrete time step *n*. Whereas, *d*_*y*_ and *d*_*u*_ are the orders of memory in the output and input, respectively, and they need to obey to: *d*_*y*_ ≥ 1 and *d*_*u*_ ≥ 1 and *d*_*u*_ ≥ *d*_*y*_. This means that the future value of the dependent output variable (*y*(*n* + 1)) is regressed on previous values of the output and previous values of an independent (exogenous) input. To implement the function *f*, a feedforward neural network can be utilized.

**FIGURE 1:**
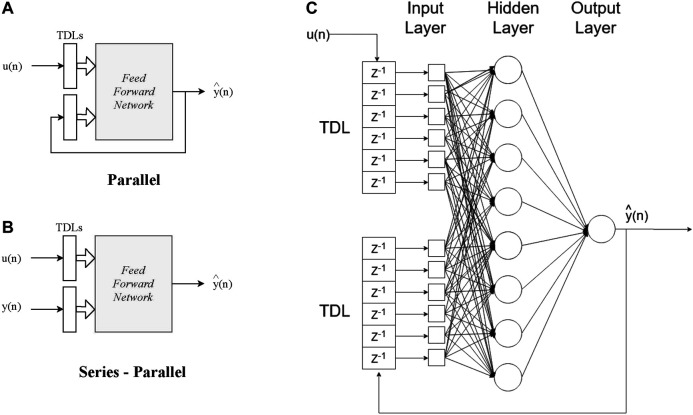
Configuration of NARX. **(A)** Parallel and **(B)** Series-Parallel modes **(C)** NARX architecture utilized in this study.

Equation 1 can be given in vector form as:

y(n+1)=f[y(n),u(n)] (2)

where the vectors *y*(*n*) and *u*(*n*) can be termed as the output and input regressors, respectively. Generally, there are two configurations of the NARX neural network model training procedure, the parallel mode (also known as open loop, shown in Figure 1A) and the series-parallel mode (also known as open loop, shown in Figure 1B). In the open loop case, the output regressor (*y*(*n*)) is formed only by output values of the actual system to be modelled. On the other hand, in the closed loop case, the estimated outputs by the network are used to form the output regressor. Typically, the open loop mode is utilized during the training of the network, given that the real output values of the actual system are known a priori. Consequently, the input to the feed-forward network is more accurate and conventional back-propagation can be used for training, resulting to better performance. When the network is used for prediction, the closed loop case can be utilized to provide long term predictions. A more detailed configuration is depicted in Figure 1C.

Here, a NARX network was implemented for the prediction of the voltage of a MFC that can be employed as an energy source for soft robots. For the training and the testing phase of the network, the open loop form was used, as described in the previous, given that the dataset used is containing the output values of actual MFCs developed in the lab (more details in Section 4). Moreover, one independent (exogenous) input was defined, namely the volume of feedstock inserted to the MFC chamber during that time interval.

## 4 Methods

**FIGURE 2:**
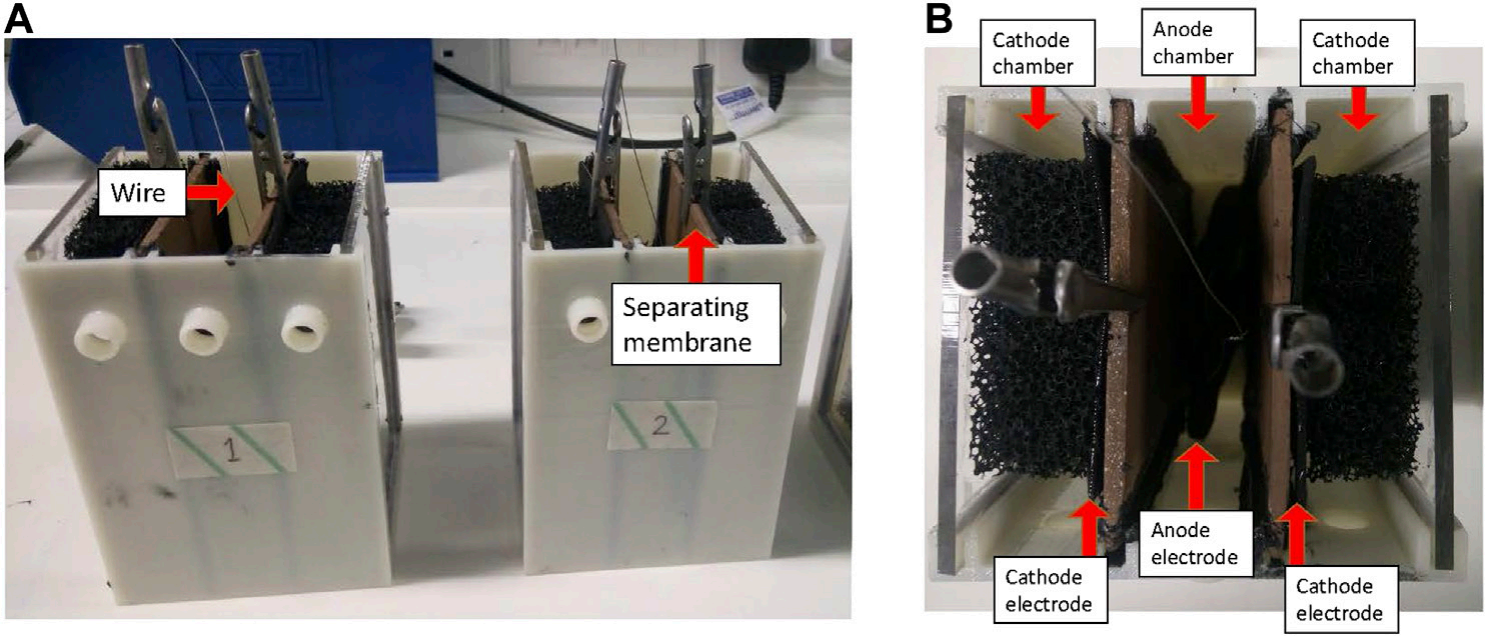
Photographs of the MFCs used in laboratory experiments. **(A)** Front view and **(B)** top view.

To evaluate the efficiency of a NARX network in predicting the voltage output of MFCs, a dataset was constructed after the development of MFCs in the lab. The MFCs under study are constructed via 3D printing with ABS (Acrylonitrile Butadiene Styrene). Both anode and a pair of cathode chambers have a volume of 165 ml each. The anode electrode is made from activated carbon modified carbon veil sheets with dimensions of, (9 × 30) = 270 cm^2^ and with 5 sheets used total dimensions of 270 × 5 = 1350 cm^2^. Whereas, double cathode electrodes are made from hot-pressed activated carbon onto stainless steel mesh backbone, with dimensions of (6 × 11) × 2 = 132 cm^2^ (each cathode size of 66 cm^2^). The membrane separating the anode and cathode chambers is a custom-made ceramic sheet (product no.: 366, Goerg & Schneider, Siershahn, Germany) of 7.5 cm width, 11 cm height and wall thickness of 3 mm. Open-to-air and partially submerged to 80 ml tap water (added to each cathode chamber of 3D printed boxes) type cathodes were used. The final 3D printed MFCs are depicted in Figure 2. MFCs were inoculated with 1:1 mixture of human urine and activated sewage sludge (Wessex Water Scientific Laboratory, Cam Valley, Saltford, UK) enriched with 1% tryptone, 0.5% yeast extract and 0.5% sodium acetate. The MFCs were batch fed with human urine and initially loaded with an external resistance of 500 Ω. On day 2, MFCs were inoculated with the same inoculum again, and the external load changed to 200 Ω. After 4 days of the second inoculation the voltage measurements were used. A multichannel Agilent recorder data logger (LXI 34972A data acquisition/Switch unit) was used to continuously monitor the MFC voltage, by taking measurement every 5 min.

The dataset was compiled with pairs of voltage output and feedstock volume inserted, with time stamps for each pair, namely two time series. Two identical MFCs were developed and the same procedure was used for both of them to enhance the robustness of the prediction model. The measurements have lasted for 14 days and consecutive samples were 5 min apart (resulting to 4032 data points for each MFC, in total 8064). The dataset used was extracted from these data points by taking 1 h intervals instead of 5 min. This was decided based on two main reasons. First, the application in mind is the implementation of soft robots equipped with MFCs, so the response of MFCs -being based on biological processes- is better positioned at hourly intervals rather than 5 min and the frequent monitoring of the MFC output in the field will cost even more energy dissipation overheads. Moreover, the use of hourly intervals will reduce the risk of over-fitting the neural network model, given the fact that the feeding instances are only two in the 14 days long experiments. To implement the NARX model training and testing, the Deep Learning Toolbox of Matlab 2019b was used (Mathworks, 2020).

**FIGURE 3:**
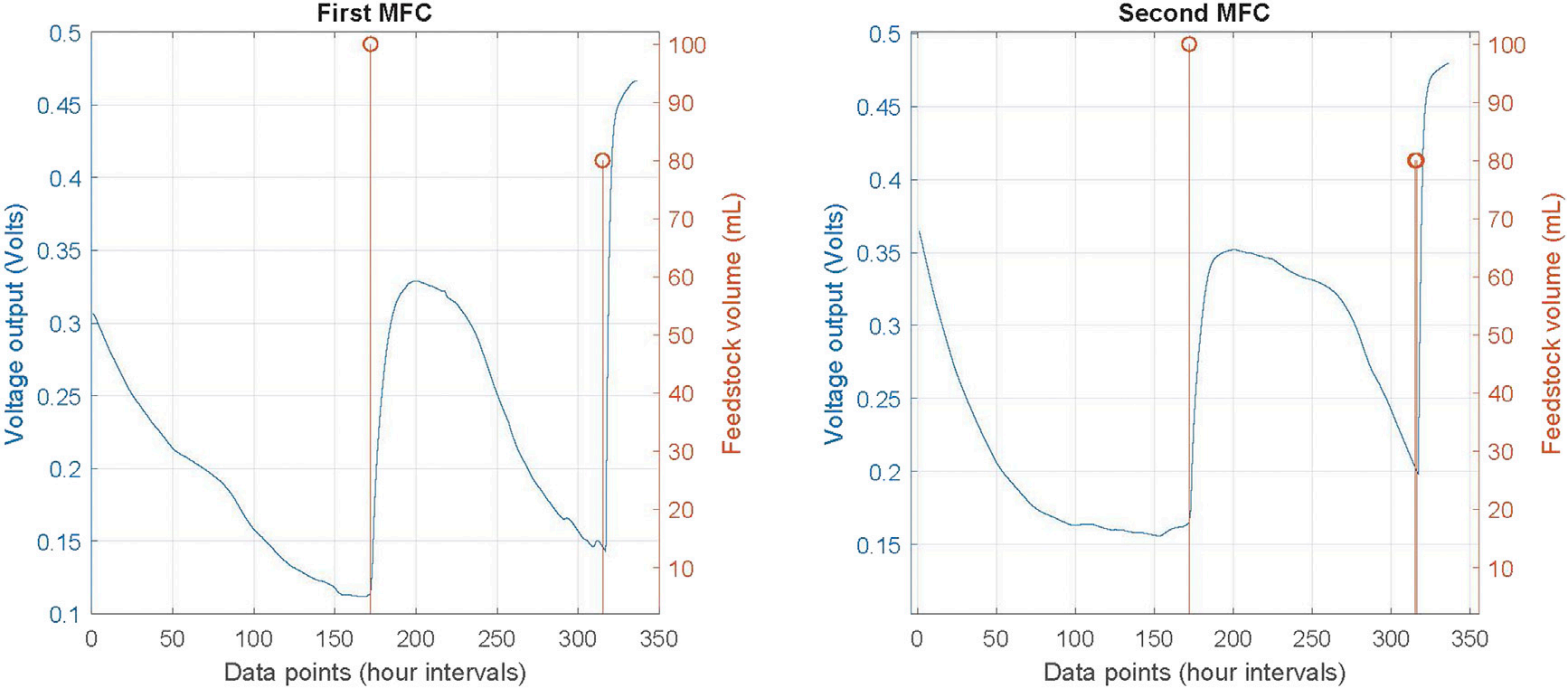
Dataset of voltage output and feedstock added of both MFCs.

As mentioned before, the dataset was filtered to make the time intervals between data points 1 h. The critical time intervals, when feedstock was added, were maintained in all cases. This resulted to a dataset of 336 data points for each MFC. The voltage outputs for both MFCs and the feedstock added are presented in Figure 3. The initial voltage output of both MFCs after the inoculation phase is c. 0.3 and 0.35 V (as can be observed based on the left *y*-axis and the blue line). Then, voltage is reducing in an exponentially manner, until the first feeding instance of 100 ml (at data point or hourly interval of 172, as can be observed based in the right *y*-axis and the orange line). After that the voltage sharply increases, reaches a plateau and then exponentially decreases again. The second feeding instance of 80 ml is occurring at data point 316, followed by a similar behaviour of both MFCs.

For the training process the dataset from measurements of the first MFC was randomly divided into three subsets. The training set was defined at 80% (i.e. 269 data points), the validation set was defined at 15% (i.e. 50 data points), while the test set at 5% (i.e. 17 data points). Note that the test set was determined at a very low percentage as it does not affect the training procedure, but it is just an independent measure of the performance of the network. Moreover, the network was then tested upon the measurements acquired from the second MFC to certify its robustness.

The NARX network was set in the open loop mode for the training. The hidden layer was assigned with eight neurons and the order of memory (or delay) for both input and output was set to 6 (*d*_*u*_ = *d*_*y*_ = 6 on Eq. 1), as illustrated in Figure 1C. The training process was implemented with the Levenberg-Marquardt backpropagation (Hagan and Menhaj, 1994; Hagan et al., 1996), as developed under the Matlab 2019b Deep Learning Toolbox (Mathworks, 2020).

## 5 Results and Discussion

**FIGURE 4:**
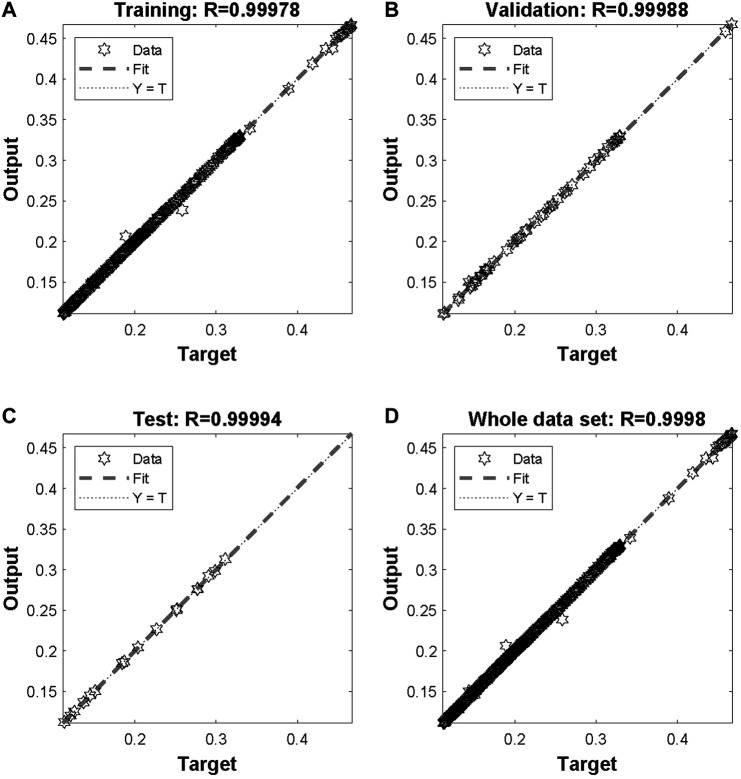
Regression plots showing network results compared with targets for **(A)** training, **(B)** validation, **(C)** test and **(D)** whole data set.

**FIGURE 5:**
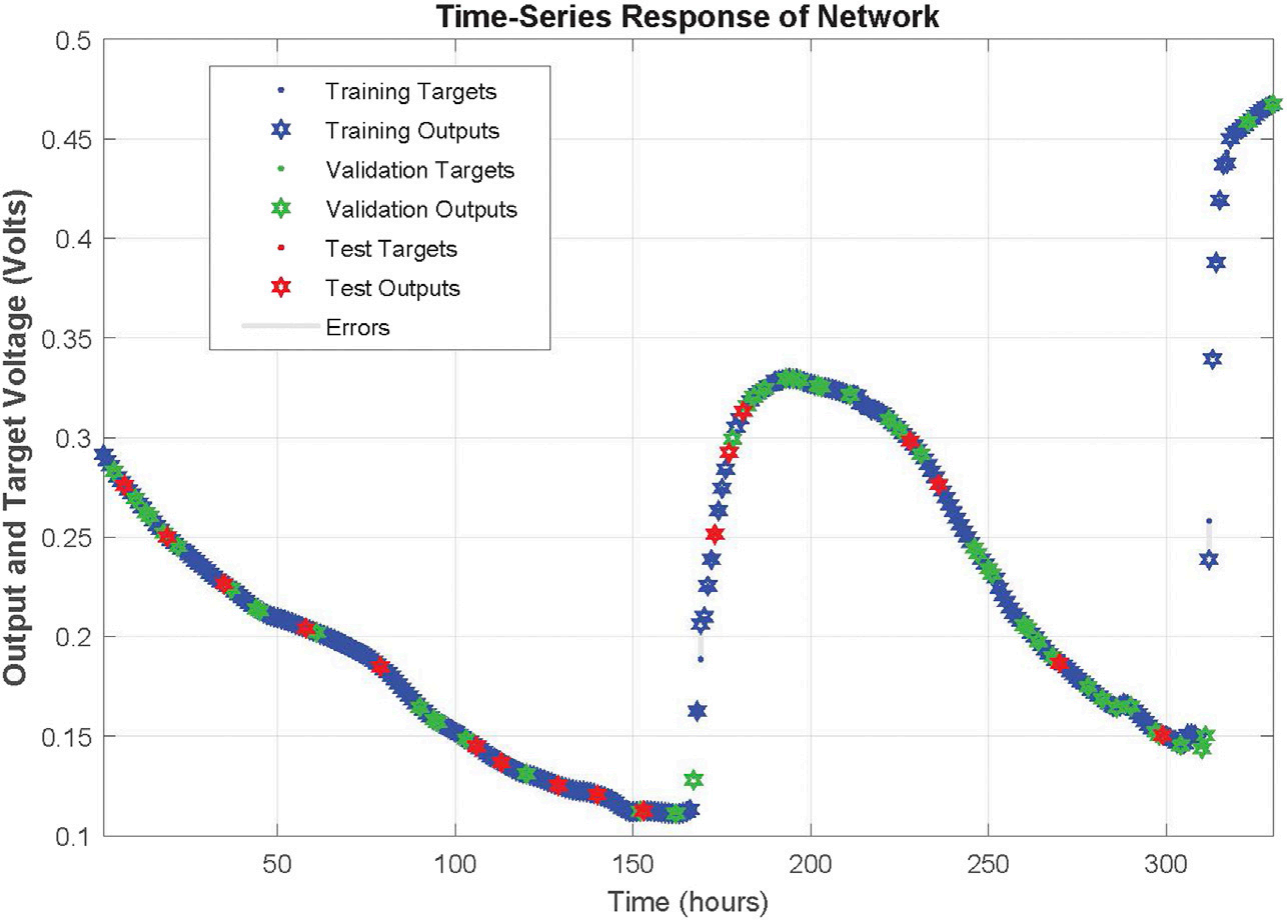
Response of NARX on training, validation and testing dataset time-series of first MFC.

**FIGURE 6:**
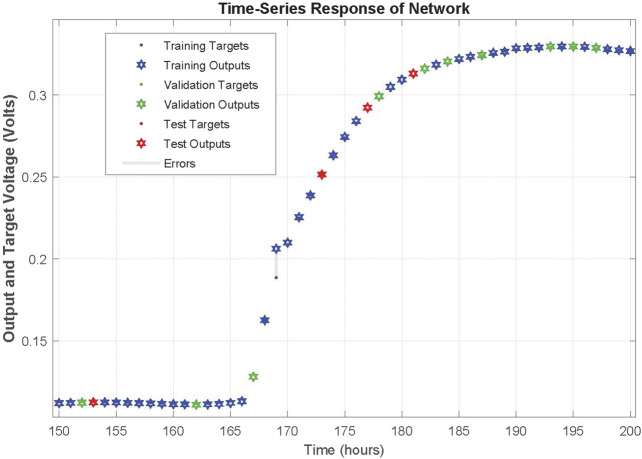
Zoomed in response of NARX on training, validation and testing dataset time-series of first MFC at the first refilling of feedstock.

The network was trained for 25 epochs and the behaviour of the network can be realized by the regression plots illustrated in Figure 4. More specifically, all data points seem to be close enough to the 45° that denotes the perfect fit between network output and targeted real values. The correlation coefficient (*R*) for training, validation, test and the whole data set are 0.99978, 0.99988, 0.99994 and 0.9998, respectively. The response of the network during training is illustrated in Figure 5 and a close up to the first feeding interval is illustrated in Figure 6. The target (actual) data points and the network outputs are depicted with appropriate encoding in these figures for the training, validation and testing sets.

**FIGURE 7:**
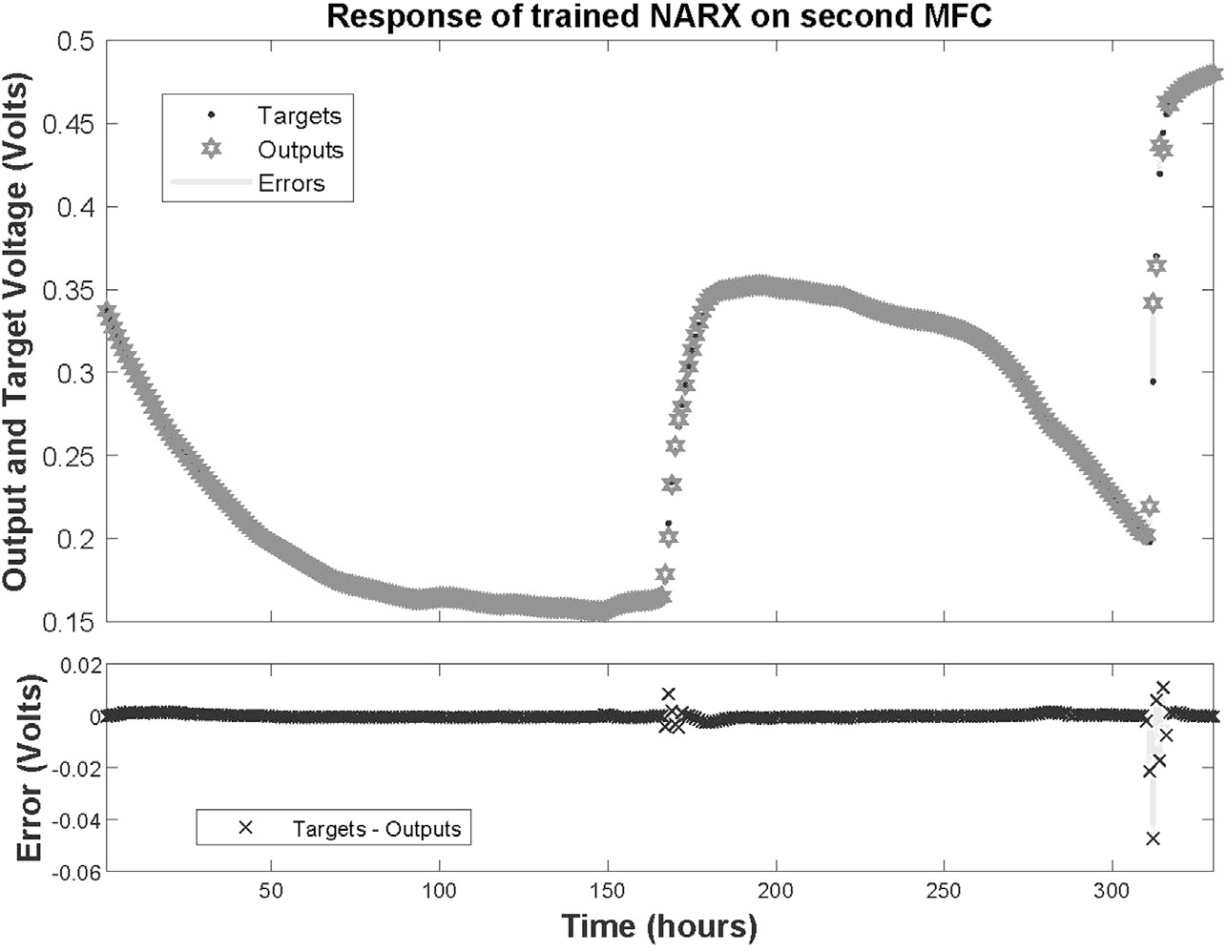
Response of trained NARX on dataset time-series of second MFC and associated errors.

**FIGURE 8:**
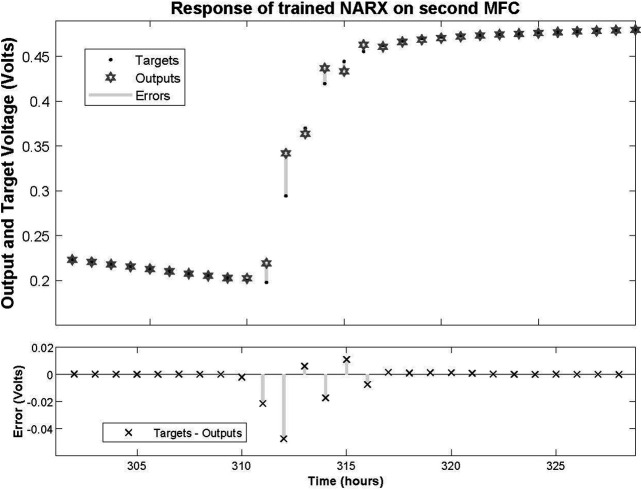
Zoomed in response of trained NARX on dataset time-series of second MFC at the second refilling of feedstock and associated errors.

After training the NARX network on the dataset obtained by the first MFC measurements, it was tested on a dataset not included in the training procedure; namely, the measurements of the second MFC. The resulted response that the open loop NARX provided with the associated errors are depicted in Figures 7, 8. The performance of the network can be characterized by a Mean Square Error (MSE) of 1.049 × 10^−5^ and *R* of 0.999317. This reveals that despite the fact that the network was trained on a limited dataset of just one MFC behaviour, when deployed to predict a time-series never processed before, it performed almost perfectly.

Note that despite the fact that both MFCs are fabricated with identical procedures, their behaviour after inoculations and refilling feedstocks are significantly different. This observation enhances the suggestion that MFCs should be approximated with nonlinear modeling techniques, i.e. the NARX model proposed here.

## 6 Conclusion

A novel alternative of toxic materials used for energy storage on soft robotics can be MFCs. Their ability to be developed by biodegradable and bio-compatible materials enable the entire soft robot entity to not burden the environment. However, MFCs are not easily modeled nor their outputs can be exactly replicated. As a result, the use of ML and, in specific, NARX model for the prediction of their outputs was proposed here. By using this smart method of tracking the MFCs outputs and predicting the behaviour after new feedstock is added, will enhance the effective applicability of MFCs as energy providers for soft robotics.

The choice of NARX model was based on the fact that they are easy to implement and can be transformed between open loop and closed loop modes based on the application phase. More specifically, open loop allows for more accurate training, while closed loop networks enable multistep predictions. In other words, closed loop mode continues to predict when external feedback is missing or unavailable at the instant needed, by using internal feedback. The same network can alternate between open and closed loop form, depending on the availability of the last time interval reading availability.

Future work will include implementation of the model in energy-autonomous robots to evaluate its efficacy for determining feed times, and the efficiency of this mode of feed scheduling compared to sampling the MFC voltage with higher frequency. This will be employed in the future development of self-feeding soft robots.

## Data Availability

The raw data supporting the conclusions of this article will be made available by the authors, without undue reservation.

## References

[B1] de Ramón-Fernández A. Salar-García M. J. Ruiz Fernández D. Greenman J. Ieropoulos I. A. (2020). Evaluation of artificial neural network algorithms for predicting the effect of the urine flow rate on the power performance of microbial fuel cells. Energy 213, 118806. 10.1016/j.energy.2020.118806 33335352 PMC7695679

[B2] Esfandyari M. Fanaei M. A. Gheshlaghi R. Mahdavi M. A. (2016). Neural network and neuro-fuzzy modeling to investigate the power density and columbic efficiency of microbial fuel cell. J. Taiwan Ins. Chem. Engin. 58, 84–91. 10.1016/j.jtice.2015.06.005

[B3] Feng Y. Kayode O. Harper W. F. Jr (2013). Using microbial fuel cell output metrics and nonlinear modeling techniques for smart biosensing. Sci. total Environ. 449, 223–228. 10.1016/j.scitotenv.2013.01.004 23428752

[B4] Garg A. Vijayaraghavan V. Mahapatra S. S. Tai K. Wong C. H. (2014). Performance evaluation of microbial fuel cell by artificial intelligence methods. Expert Systems with Applications 41, 1389–1399. 10.1016/j.eswa.2013.08.038

[B5] Garrad M. Soter G. Conn A. Hauser H. Rossiter J. (2019). A soft matter computer for soft robots. Sci Robot 4, eaaw6060. 10.1126/scirobotics.aaw6060 33137781

[B6] Ghasemi M. Nassef A. M. Al-Dhaifallah M. Rezk H. (2020). Performance improvement of microbial fuel cell through artificial intelligence. Int. J. Energy Res. 45, 5484. 10.1002/er.5484

[B7] Hagan M. Demuth H. Beale M. de Jesús O. (1996). Neural network design. Boston, MA: PWS publishing company.

[B8] Hagan M. T. Menhaj M. B. (1994). Training feedforward networks with the marquardt algorithm. IEEE Trans Neural Netw 5, 989–993. 10.1109/72.329697 18267874

[B9] He Y.-J. Ma Z.-F. (2016). A data-driven Gaussian process regression model for two-chamber microbial fuel cells. Fuel Cells 16, 365–376. 10.1002/fuce.201500109

[B10] Ieropoulos I. Greenman J. Melhuish C. Horsfield I., (2010). Ecobot-iii-a robot with guts. ALIFE. 733, e40.

[B11] Ieropoulos I. Greenman J. Melhuish C. (2003). Imitating metabolism: energy autonomy in biologically inspired robots. Proc. AISB 3, 191–194.

[B12] Ieropoulos I. Melhuish C. Greenman J. Horsfield I. (2005). Ecobot-ii: an artificial agent with a natural metabolism. International Journal of Advanced Robotic Systems 2, 31. 10.5772/5777

[B13] Ismail Z. Z. Al-wared A. I. Jaeel A. J. (2017). Recourse recovery of bioenergy from cellulosic material in a microbial fuel cell fed with giant reed-loaded wastewater. Biofuels 10, 1–9. 10.1080/17597269.2017.1409057

[B14] Jadhav D. A. Carmona-Martínez A. A. Chendake A. D. Pandit S. Pant D. (2021). Modeling and optimization strategies towards performance enhancement of microbial fuel cells, Bioresour. Technol. 320, 124256. 10.1016/j.biortech.2020.124256 33120058

[B15] Jaeel A. J. Al-wared A. I. Ismail Z. Z. (2016). Prediction of sustainable electricity generation in microbial fuel cell by neural network: effect of anode angle with respect to flow direction. Journal of Electroanalytical Chemistry 767, 56–62. 10.1016/j.jelechem.2016.02.015

[B16] Lesnik K. L. Liu H. (2017). Predicting microbial fuel cell biofilm communities and bioreactor performance using artificial neural networks. Environ. Sci. Technol. 51, 10881–10892. 10.1021/acs.est.7b01413 28812881

[B17] Li S. Wang K. (2015). Fluidic origami with embedded pressure dependent multi-stability: a plant inspired innovation. J R Soc Interface 12, 20150639. 10.1098/rsif.2015.0639 26400197 PMC4614500

[B18] Lin T. Horne B. G. Tino P. Giles C. L. (1996). Learning long-term dependencies in narx recurrent neural networks. IEEE Trans Neural Netw 7, 1329–1338. 10.1109/72.548162 18263528

[B19] Méhes G. Roy A. Strakosas X. Berggren M. Stavrinidou E. Simon D. T. (2020). Organic microbial electrochemical transistor monitoring extracellular electron transfer. Adv Sci (Weinh) 7, 2000641. 10.1002/advs.202000641 32775155 PMC7404149

[B20] Mathworks (2020). MATLAB. Avaialble at: https://uk.mathworks.com/help/deeplearning/ref/trainlm.html. [Accessed October 15, 2020]

[B21] Menezes J. M. P. Barreto G. A. (2008). Long-term time series prediction with the narx network: an empirical evaluation. Neurocomputing 71, 3335–3343. 10.1016/j.neucom.2008.01.030

[B22] Pastor F. Gandarias J. M. García-Cerezo A. J. Gómez-de-Gabriel J. M. (2019). Using 3d convolutional neural networks for tactile object recognition with robotic palpation. Sensors 19, 5356. 10.3390/s19245356 PMC6960774 31817320

[B23] Philamore H. Ieropoulos I. Stinchcombe A. Rossiter J. (2016). Toward energetically autonomous foraging soft robots. Soft Robotics 3, 186–197. 10.1089/soro.2016.0020

[B24] Philamore H. Rossiter J. Ieropoulos I. (2015a). “An energetically-autonomous robotic tadpole with single membrane stomach and tail,” in Conference on biomimetic and biohybrid systems. New York: Springer, 366–378.

[B25] Philamore H. Rossiter J. Stinchcombe A. Ieropoulos I. (2015b). “Row-bot: an energetically autonomous artificial water boatman,” in 2015 IEEE/RSJ international conference on intelligent robots and systems (IROS), Hamburg, Germany, September 28–October 2, 2015 (IEEE), 3888–3893. 10.1109/IROS.2015.7353924

[B26] Picioreanu C. van Loosdrecht M. C. Katuri K. P. Scott K. Head I. M. (2008). Mathematical model for microbial fuel cells with anodic biofilms and anaerobic digestion. Water Sci. Technol. 57, 965–971. 10.2166/wst.2008.095 18441420

[B27] Picioreanu C. van Loosdrecht M. C. Curtis T. P. Scott K. (2010). Model based evaluation of the effect of ph and electrode geometry on microbial fuel cell performance. Bioelectrochemistry 78, 8–24. 10.1016/j.bioelechem.2009.04.009 19523880

[B28] Pinto R. Srinivasan B. Manuel M. F. Tartakovsky B. (2010). A two-population bio-electrochemical model of a microbial fuel cell. Bioresour. Technol. 101, 5256–5265. 10.1016/j.biortech.2010.01.122 20171879

[B29] Preston D. J. Rothemund P. Jiang H. J. Nemitz M. P. Rawson J. Suo Z. Whitesides G. M. (2019). Digital logic for soft devices. Proc Natl Acad Sci U S A 116, 7750–7759. 10.1073/pnas.1820672116 30923120 PMC6475414

[B30] Rossiter J. Winfield J. Ieropoulos I. (2017). “Eating, drinking, living, dying and decaying soft robots,” in Soft robotics: trends, applications and challenges, New York: Springer, 95–101.

[B31] Rossiter J. Winfield J. Ieropoulos I. (2016). “Here today, gone tomorrow: biodegradable soft robots,” in Electroactive polymer actuators and devices (EAPAD) 2016, Bellingham, Washington: International Society for Optics and Photonics, 97981S.

[B32] Rothemund P. Ainla A. Belding L. Preston D. J. Kurihara S. Suo Z. (2018). A soft, bistable valve for autonomous control of soft actuators. Sci. Robotics 3, eaar7986. 10.1126/scirobotics.aar7986 33141749

[B33] Santoro C. Arbizzani C. Erable B. Ieropoulos I. (2017). Microbial fuel cells: from fundamentals to applications. a review. J Power Sources 356, 225–244. 10.1016/j.jpowsour.2017.03.109 28717261 PMC5465942

[B34] Slate A. J. Whitehead K. A. Brownson D. A. C. Banks C. E. (2019). Microbial fuel cells: an overview of current technology. Renewable and Sustainable Energy Reviews 101, 60–81. 10.1016/j.rser.2018.09.044

[B35] Song Y. Panas R. M. Chizari S. Shaw L. A. Jackson J. A. Hopkins J. B. (2019). Additively manufacturable micro-mechanical logic gates. Nat. Commun. 10, 1–6. 10.1038/s41467-019-08678-0 30787283 PMC6382908

[B36] Tardast A. Rahimnejad M. Najafpour G. Ghoreyshi A. Premier G. C. Bakeri G. (2014). Use of artificial neural network for the prediction of bioelectricity production in a membrane less microbial fuel cell. Fuel 117, 697–703. 10.1016/j.fuel.2013.09.047

[B37] Tardast A. Rahimnejad M. Najafpour G. Pirzade K. Mokhtarian N. (2012). Prediction of bioelectricity production by neural network. J. Biotechnol. Pharm. Res. 3, 62–68.

[B38] Teuscher C. (2014). Unconventional computing catechism. Front. Robotics AI 1, 10. 10.3389/frobt.2014.00010

[B39] Thuruthel T. G. Ansari Y. Falotico E. Laschi C. (2018). Control strategies for soft robotic manipulators: a survey. Soft robotics 5, 149–163. 10.1089/soro.2017.0007 29297756

[B40] Thuruthel T. G. Falotico E. Cianchetti M. Laschi C. (2016). Learning global inverse kinematics solutions for a continuum robot. in Symposium on robot design, dynamics and control. New York: Springer), 47–54.

[B41] Thuruthel T. G. Falotico E. Renda F. Laschi C. (2019). Model-based reinforcement learning for closed-loop dynamic control of soft robotic manipulators. IEEE Trans. Robot. 35, 124–134. 10.1109/tro.2018.2878318

[B42] Tsompanas M. A. Adamatzky A. Ieropoulos I. Phillips N. Sirakoulis G. C. Greenman J. (2017a). Cellular non-linear network model of microbial fuel cell. BioSystems 156–157, 53–62. 10.1016/j.biosystems.2017.04.003 28428117

[B43] Tsompanas M. I. Adamatzky A. Sirakoulis G. C. Greenman J. Ieropoulos I. (2017b). Towards implementation of cellular automata in microbial fuel cells. PloS one 12, e0177528. 10.1371/journal.pone.0177528 28498871 PMC5428934

[B44] Tsompanas M.-A. Adamatzky A. Ieropoulos I. Phillips N. W. Sirakoulis G. C. Greenman J. (2018). Modelling microbial fuel cells using lattice Boltzmann methods. IEEE/ACM Trans. Comput. Biol. Bioinformatics 16, 2035–2045. 10.1109/TCBB.2018.2831223 29994029

[B45] Tsompanas M.-A. You J. Wallis L. Greenman J. Ieropoulos I. (2019). Artificial neural network simulating microbial fuel cells with different membrane materials and electrode configurations. Journal of Power Sources 436, 226832. 10.1016/j.jpowsour.2019.226832

[B46] Wang J. Wang Q. Zhou J. Wang X. Cheng L. (2018). Operation space design of microbial fuel cells combined anaerobic-anoxic-oxic process based on support vector regression inverse model. Engineering Applications of Artificial Intelligence 72, 340–349. 10.1016/j.engappai.2018.04.005

[B47] Wilson E. D Assaf T. Pearson M. J Rossiter J. M. Anderson S. R. Porrill J. (2016). Cerebellar-inspired algorithm for adaptive control of nonlinear dielectric elastomer-based artificial muscle. J R Soc Interface 13, 20160547. 10.1098/rsif.2016.0547 27655667 PMC5046955

[B48] Winfield J. Chambers L. D. Rossiter J. Greenman J. Ieropoulos I. (2015b). Urine-activated origami microbial fuel cells to signal proof of life. J. Mater. Chem. A 3, 7058–7065. 10.1039/c5ta00687b

[B49] Winfield J Chambers LD Rossiter J Ieropoulos I (2013a). Comparing the short and long term stability of biodegradable, ceramic and cation exchange membranes in microbial fuel cells. Bioresour. Technol. 148, 480–486. 10.1016/j.biortech.2013.08.163 24077158

[B50] Winfield J. Chambers L. D. Stinchcombe A. Rossiter J. Ieropoulos I. (2014). The power of glove: soft microbial fuel cell for low-power electronics. Journal of Power Sources 249, 327–332. 10.1016/j.jpowsour.2013.10.096

[B51] Winfield J. Chambers L. Rossiter J. Stinchcombe A. Walter X. A. Greenman J. (2015a). Fade to green: a biodegradable stack of microbial fuel cells. ChemSusChem 8, 2705. 10.1002/cssc.201500431 26212495

[B52] Winfield J. Ieropoulos I. Rossiter J. Greenman J. Patton D. (2013b). Biodegradation and proton exchange using natural rubber in microbial fuel cells. Biodegradation 24, 733–739. 10.1007/s10532-013-9621-x 23361125

